# A Pharmacoepidemiological Study of Myocarditis and Pericarditis Following the First Dose of mRNA COVID-19 Vaccine in Europe

**DOI:** 10.3390/microorganisms11051099

**Published:** 2023-04-22

**Authors:** Joana Tome, Logan T. Cowan, Isaac Chun-Hai Fung

**Affiliations:** Department of Biostatistics, Epidemiology, and Environmental Health Sciences, Jiann-Ping Hsu College of Public Health, Georgia Southern University, Statesboro, GA 30458, USA

**Keywords:** COVID-19, adverse drug reaction, myocarditis, pericarditis, COVID-19 mRNA vaccine

## Abstract

This study assessed the myocarditis and pericarditis reporting rate of the first dose of mRNA COVID-19 vaccines in Europe. Myocarditis and pericarditis data pertinent to mRNA COVID-19 vaccines (1 January 2021–11 February 2022) from EudraVigilance database were combined with European Centre for Disease Prevention and Control (ECDC)’s vaccination tracker data. The reporting rate was expressed as events (occurring within 28 days of the first dose) per 1 million individuals vaccinated. An observed-to-expected (OE) analysis quantified excess risk for myocarditis or pericarditis following the first mRNA COVID-19 vaccination. The reporting rate of myocarditis per 1 million individuals vaccinated was 17.27 (95% CI, 16.34–18.26) for CX-024414 and 8.44 (95% CI, 8.18–8.70) for TOZINAMERAN; and of pericarditis, 9.76 (95% CI, 9.06–10.51) for CX-024414 and 5.79 (95% CI, 5.56–6.01) for TOZINAMERAN. Both vaccines produced a myocarditis standardized morbidity ratio (SMR) > 1, with the CX-024414 vaccine having a greater SMR than TOZINAMERAN. Regarding TOZINAMERAN, SMR for pericarditis was >1 when considering the lowest background incidence, but <1 when considering the highest background incidence. Our results suggest an excess risk of myocarditis following the first dose of the mRNA COVID-19 vaccine, but the relationship between pericarditis and the mRNA COVID-19 vaccine remains unclear.

## 1. Introduction

Myocarditis (inflammation of the heart muscle) and pericarditis (inflammation of the heart’s outer lining) following immunizations such as smallpox, influenza, hepatitis B, or other vaccines have previously been reported as a rare side effect [1,2,3]. Depending on the source, the annual incidence of myocarditis and pericarditis in the European Economic Area (EEA) has ranged from 1 to 10 in 100,000 people [4]. The European Medicines Agency (EMA)’s safety committee, the Pharmacovigilance Risk Assessment Committee (PRAC), started reviewing cases of myocarditis and pericarditis following Pfizer-BioNTech (TOZINAMERAN) and Moderna (CX-024414) coronavirus disease 2019 (COVID-19) vaccination since April 2021. Increased rates of cases of myocarditis and pericarditis following messenger RNA (mRNA) COVID-19 vaccination were also noticed in the United States (US) [5,6] and Canada [7].

TOZINAMERAN and CX-024414, the two mRNA vaccines being used in the European Union (EU)/EEA, contain an mRNA molecule with instructions for producing the spike protein from severe acute respiratory syndrome coronavirus 2 (SARS-CoV-2) that stimulates the body’s immune response [4]. There are different biological mechanisms that can explain the possible association between mRNA COVID-19 vaccination and the occurrence of myocarditis and pericarditis. Based on preliminary evidence from adult trials with mRNA vaccinations, it is rare but possible that antibody responses to mRNA vaccines can become very high, causing cardiac inflammation [8]. One other mechanism is the activation of anti-idiotype cross-reactive antibody-mediated cytokine expression in the myocardium, as well as abnormal apoptosis, which can result in myocardial and pericardial inflammation [9]. The mRNA vaccines may possibly trigger a non-specific innate inflammatory response or a molecular mimicry mechanism between the viral spike protein and an unknown cardiac protein [10]. It may be a side effect of the inflammatory process created by vaccination, but not related to the viral spike protein [11]. The last explanation is that vaccinations’ potent immunogenic RNA can have a bystander or adjuvant consequence on the heart [12].

### Literature Review

Although the occurrence of myocarditis and pericarditis following SARS-CoV-2 infection seems to be more frequent and serious [13,14,15], assessing the risk of myocarditis and pericarditis after COVID-19 mRNA immunization has its own importance.

Studies of the possible occurrence of myocarditis and pericarditis after immunization with the mRNA COVID-19 vaccines have been performed worldwide. A short review of existing literature related to the relationship between COVID-19 mRNA vaccines and the occurrence of myocarditis and pericarditis is provided below.

Bozkurt, Kamat, and Hotez [2] observed the reports posted during 2021 by Vaccine Adverse Event Reporting System (a passive reporting system of the Centers for Disease Control and Prevention (CDC)), the CDC’s Vaccine Safety Datalink (VSD), and the US Department of Defense, and found that the majority of the reported cases were male, 12 to 39 years old, and had chest pain around two to three days after the second vaccination dose. From these reports, it was supposed that myocarditis/pericarditis rates were around 12.6 cases per million doses of second-dose mRNA vaccines in the US [2]. The authors suggested that myocarditis caused by mRNA vaccination was different from the typical lymphocytic myocarditis associated with other viral myocarditis presentations, and the male predominance may be related to sex hormone differences in immune response as well as underdiagnosis of cardiovascular disease among women [2].

Diaz et al. [16] reported rare cases of myocarditis or pericarditis following SARS-CoV-2 vaccination from late 2020 through 25 May 2021, detected in 40 hospitals in Washington, Oregon, Montana, and Los Angeles County, California, which utilized an identical electronic medical record (EMR) system and were affiliated with the Providence health system. Among 20 myocarditis patients, 75% were male, the median age was 36 years (interquartile range (IQR), 26–48 years), 20% developed symptoms following the first dose, while 80% of the patients developed symptoms following the second dose, and the median onset was 3.5 days (IQR, 3.0–10.8 days) after vaccination [16]. Among 15 patients with pericarditis, the median age of the participant was 59 (IQR, 46–69 years), 40.5% developed symptoms after the first dose, while 59.5% after the second dose, and the median onset time was around 20 days (IQR, 6.0–41.0 days) [16]. Possible study limitations are missed cases and diagnoses outside the care settings (which can underestimate the incidence) and inaccurate information from EMR.

Gargano et al. [17] reported VAERS detected myocarditis cases from 29 December 2020, to 11 June 2021. The cases’ median age was 26 years (range = 12–94 years), with a median symptom onset of three days after vaccination, and 76% of cases occurred after receiving the second dose [17]. Among 1212 identified cases with known sex, 923 were males, where those aged 12–24 years had the highest myocarditis reporting rates. The authors reported cases from VSD too, and although numbers were too small to show rates in all subgroups by age, they saw an increased risk of myocarditis 7 days after receiving a first or a second dose of both mRNA COVID-19 vaccines.

Kim et al. [18] presented a series of seven patients with acute myocarditis between the 1 February 2021 and 30 April 2021, identified at Duke University Medical Center with finalized clinical cardiac magnetic resonance imaging (MRI) reports. Four cases occurred within 5 days of the second dose of mRNA vaccine (two received the Pfizer vaccine, and the others received the Moderna vaccine), and three of them were younger male individuals (23–36 years old) [18]. None of the identified cases had a viral symptom or COVID-19 infection, and the myocarditis diagnosis was straightforward [18]. However, the researchers were able to locate only cases with severe symptoms who sought medical attention.

Starekova et al. [19], with a retrospective study, reported cardiac MRI findings of myocarditis in five patients who lately received mRNA COVID-19 vaccination between 1 January through 25 May 2021, in Wisconsin. Four of these patients were male, and the participants ranged in age from 17 to 38 years old [19]. Three patients received the second dose of the Pfizer-BioNTech vaccine 2 to 3 days before symptoms onset, while the other two patients received the second dose of Moderna vaccine 3 days before the start of the symptoms [19]. There was no previous history of COVID-19 infection among five cases or other infections, and there was a clear temporal relationship. Other patients with a history of prior COVID-19 infection were not included in the study. However, the researchers did not perform myocardial biopsy because of the mild and uncomplicated form of myocarditis, or any serological test.

Verma, Lavine, and Lin [20] reported two adult cases (a woman 45 years old and a man 42 years old) of fulminant myocarditis that had developed within 2 weeks after the COVID-19 vaccination. No test for viral genomes or autoantibodies in the tissue specimens was performed; therefore, a direct causal relationship could not be established.

Through a cross-sectional study design, Das et al. [9] summarized 29 cases (children and adults) of myocarditis and pericarditis published in the literature as of 26 June 2021. All subjects were males; four were from Italy, the rest were from the US, and 13 were 18 years older or younger [9]. Twenty-eight cases were related to the mRNA vaccination, where 10% occurred after the first dose, and the rest happened following the vaccine’s second dose [9]. The onset of symptoms mainly occurred within 3 days but ranged between 1 and 7 days [9]. Myopericarditis cases were mild and clinically resolved within a few days to a few weeks. Among the published cases, it was challenging to separate myocarditis from pericarditis, and most notably, there was no troponin data available in seven patients [9].

Marshall et al. [21] reported seven cases of clinical myocarditis or myopericarditis that developed in 14- to 19- year-old males within 4 days following the second dose of the Pfizer-BioNTech vaccine with no signs of acute SARS-CoV-2 infection. There was no evidence of other myopericarditis etiologies such as respiratory pathogens [21]. Because none of the cases were severe, no cardiac biopsy was conducted on any of them. However, given the nature of the case series, the study could not determine the incidence rate of myocarditis/myopericarditis following COVID-19 mRNA vaccination [21].

The above mentioned studies and many others may indicate that the incidence of myocarditis and pericarditis can increase following COVID-19 mRNA immunization [16,22,23,24,25]; therefore, it is critical to determine the magnitude of the association between myocarditis or pericarditis occurrence and mRNA COVID-19 vaccination in order to protect the most vulnerable population and to aid in the differentiation of the COVID-19 vaccination’s risks and benefits. Moreover, phase three clinical trials were unable to identify rare adverse events of COVID-19 vaccines because they were underpowered to detect rare events. Their detection is critical for risk-benefit analyses and informing post-vaccination clinical practice. Therefore, detecting such adverse events through pharmacovigilance post-authorization has become a global scientific priority.

To enhance risk communication that accompanies existing COVID-19 vaccination campaigns, this study aims to quantify the elevated risk, if any, of myocarditis and pericarditis 28 days following Tozinameran and CX-024414 vaccination among Europeans using publicly available data from the EU/EEA in an exploratory way. We hypothesized that myocarditis and pericarditis standardized morbidity ratios (SMRs) > 1 would result from both the mRNA COVID-19 vaccines. This study used an observed-to-expected (OE) analysis, which is a part of the quantitative pharmacovigilance toolkit for vaccines, to calculate the standardized morbidity ratio (SMR). By including medical evaluation and quantifying the unexpectedness of observing a certain number of cases, OE studies can assist in monitoring and providing insight into adverse events. Estimating the expected number of random events under the null hypothesis of no relationship with the vaccine is the fundamental principle of OE analyses. The number of cases actually reported is compared with the expected number. To run an OE analysis, we used the database on COVID-19 vaccination in the EU/EEA and EudraVigilance, which is the European database of suspected side effects by medicine or by active substance name. In EudraVigilance, each unique case relates to a single patient. The line listing complies with EU personal data privacy laws and offers a summary of individual cases involving a particular medicine or active substance and their possible adverse effects in a tabular style.

## 2. Materials and Methods

A combination of two databases was used in this pharmacoepidemiological study. The participants were Europeans from the EU/EEA countries who were eligible for the mRNA COVID-19 vaccines that were available in Europe during the 2021–2022 period. All the variables included in this analysis are named/defined as classified in their respective databases.

European Centre for Disease Prevention and Control (ECDC) has a vaccination tracker database [26] that gathers reports from the EU/EEA countries. These countries submit data to the ECDC through The European Surveillance System (TESSy) twice a week (Tuesdays and Fridays). The database contains aggregated data on the number of vaccine doses distributed by manufacturers to the EU/EEA countries, the number of first, second, additional, and unspecified doses administered to different age groups and in specific target groups, such as healthcare workers and in residents in long-term care facilities. The database includes data on the COVID-19 vaccination rollout, where each row contains the corresponding data for a certain week and country. The database is publicly available and was downloaded on 16 February 2022. From this database, we extracted the number of individuals from the EU/EEA countries who received their first dose of CX-024414 and TOZINAMERAN vaccines that also represented our exposure variable. Technically, being vaccinated with the first dose of the CX-024414 or TOZINAMERAN vaccines (including a time window from the date when the mRNA COVID-19 vaccines became eligible for vaccination in Europe till 16 February 2022) was considered as our exposure variable. In the analysis, it was used as the sum of the individuals from the EU/EEA countries who received their first dose of CX-024414 and TOZINAMERAN vaccines during the time window period, which also represented our study population size.

EudraVigilance [27] is a publicly available pharmacovigilance database of suspected adverse drug reaction (ADR) reports. Data was downloaded from EudraVigilance on 16 February 2022. The dataset contained cardiac problems related to COVID-19 vaccination using mRNA vaccines (CX-024414 and TOZINAMERAN) reported from 1 January 2021, to 11 February 2022. Among all cardiac problems, our outcomes of interest were myocarditis and pericarditis. The marketing-authorization holders (pharmaceutical corporations) and the regulatory bodies for medicines in the EEA submit the adverse events to EudraVigilance electronically. All the adverse events reported are suspected. We are unable to confirm if those agencies used the Brighton Collaboration case definition for myocarditis and pericarditis. The dataset contained the EU local number (identification number), type of report, the date when the report was received, the source of the report, age group, sex, the vaccine type, the adverse event, the duration, outcome, and seriousness of the adverse event, the duration, dose, and the route of administration of the vaccine, the type of concomitant, and the duration, dose, and the route of administration of any concomitant received by the patient. In this analysis, we used the number of myocarditis and pericarditis ADRs, the age group, sex, severity (represented by the seriousness of the adverse event), and the vaccine as the other needed variables. The ADR outcomes were treated as the sum of the number of myocarditis or pericarditis reported in the study period. Age group categories were classified as 5–11, 12–17, 18–64, 65–85 years old, more than 85 years old, and not specified. Sex was classified as female, male, and not specified. As reported in the EudraVigilance, the severity of the adverse event was classified as results in death, life-threatening, requires hospitalization/prolongation of hospitalization, results in disability/incapacity, other medically important information, and non-serious. The vaccine variable was represented by the CX-024414 and TOZINAMERAN vaccines.

Myocarditis and pericarditis reporting rates during the study period (1 January 2021, to 11 February 2022) were estimated using the formulas [28]:

- for myocarditis: $\frac{the\;number\;of\;myocarditis\;ADRs\;reported\;in\;the\;study\;period}{the\;number\;of\;individuals\;who\;received\;their\;first\;dose\;of\;vaccine\;in\;the\;study\;period}$
- for pericarditis: $\frac{the\;number\;of\;pericarditis\;ADRs\;reported\;in\;the\;study\;period}{the\;number\;of\;individuals\;who\;received\;their\;first\;dose\;of\;vaccine\;in\;the\;study\;period}$

In our EudraVigilance dataset, the information regarding the number of vaccine doses that preceded the myocarditis or pericarditis ADRs was largely missing. Therefore, an assumption was made to include all counts of the ADRs in the respective equation. It was likely that most of the reported myocarditis or pericarditis ADRs occurred after the first dose. If the individuals did not experience these ADRs after their first dose and therefore proceeded to receive their second dose, it was unlikely that these events happened after the second dose. The OE analysis was performed within 28 days post the first vaccine dose, considering the estimated lowest and highest myocarditis and pericarditis background incidence rates reported by the EMA [4]. The background incidence rate represented the number of new cases naturally occurring in the EEA, expressed as person-time, and was estimated for the EEA population before it was exposed to any of the mRNA COVID-19 vaccines. The person-time at risk was calculated as the number of first doses × (28/365.2425) × (1/100,000) [28]. The expected number of cases of myocarditis or pericarditis within 28 days of vaccination was calculated as the number of first doses × (28/365.2425) × (1/100,000) × background incidence. The OE ratio was expressed as an SMR with a 95% CI [28].

We ran a disproportionality analysis for our specified vaccines and adverse event combinations. Information Component (IC), a Bayesian statistic, was used as representative measure of disproportionality. It was defined as:

$$\mathrm{IC}=\log_{2}(\frac{O+0.5}{E+0.5})$$

where *O* are the observed and *E* are the expected cases.

The OE analysis was performed using the ‘epiR’ package in R version 4.0.3 (R Core Team, R Foundation for Statistical Computing, Vienna, Austria).

Only publicly available data were used in this study. This study was determined by Georgia Southern University’s Institution Review Board (IRB) (H20364) to be exempt from full review under the G8 exemption category (Non-human subjects determination): This project does not involve obtaining information about living individuals or does not have direct interaction or intervention with individuals or their personal data so is not defined as human subjects research under human subjects regulations. As well, no patient or public was involved in the study design or the data collection, analysis, or interpretation procedures.

## 3. Results

In the study period, there were 73,466,253 people in the EU who received the first dose of the CX-024414 vaccine and 484,402,251 who received their first dose of the TOZINAMERAN vaccine. In the same period, there were 9078 and 38,297 individuals in EU/EEA countries reported with adverse cardiac problems following the administration of the first dose of CX-024414 and the TOZINAMERAN vaccine, respectively. Of these reports, there were 1269 and 4087 myocarditis events reported for CX-024414, and for TOZINAMERAN respectively (Table 1), and 717 and 2803 pericarditis events for CX-024414 and TOZINAMERAN, respectively (Table 2). Table 3 presented the severity of myocarditis and pericarditis reported in the EudraVigilance dataset. The reporting rate of myocarditis (occurring within 28 days) per 1 million individuals vaccinated in the study period was 17.27 (95% CI, 16.34–18.26) for the CX-024414 vaccine and 8.44 (95% CI, 8.18–8.70) for TOZINAMERAN vaccine. The reporting rate for pericarditis (occurring within 28 days) per 1 million individuals vaccinated in the study period was 9.76 (95% CI, 9.06–10.51) for the CX-024414 vaccine and 5.79 (95% CI, 5.56–6.01) for TOZINAMERAN vaccine.

Table 4 and Table 5 presented the OE analyses and IC measures for both vaccines using two background incidence rates, namely 1 and 10 per 100,000 persons per year, for myocarditis and pericarditis, respectively. For myocarditis, the results showed an SMR > 1 for both vaccines, with the CX-024414 vaccine having a greater SMR than that of TOZINAMERAN (Table 4). For pericarditis, both vaccines had an SMR > 1 when considering the lowest background incidence; however, when the highest background incidence was considered, the SMR for TOZINAMERAN was <1 while that for CX-024414 was >1 (Table 5). Still, regarding pericarditis, the CX-024414 vaccine had greater SMRs than those of the TOZINAMERAN vaccine. Similar results were observed with IC. For myocarditis, the results showed an IC > 0 (where the lower bound of the 95% CI was also positive) for both vaccines with the CX-024414 vaccine having a greater IC measure than TOZINAMERAN (Table 4). For pericarditis, both vaccines had a positive IC and lower bound for the corresponding 95% CI when considering the lowest background incidence; but when the highest background incidence was considered, the IC and the lower bound of the 95% CI for the TOZINAMERAN vaccine were negative (Table 5).

## 4. Discussion

In this study, we used data from EudraVigilance to evaluate the occurrence of myocarditis and pericarditis after mRNA COVID-19 vaccination. The majority of myocarditis and pericarditis’ ADRs were reported among 18- to 64-year-olds. Myocarditis occurred mostly among male cases, while for pericarditis, there were slightly more ADRs among males for CX-024414 and slightly more ADRs among females for the TOZINAMERAN vaccine. The reporting rate of myocarditis and pericarditis (occurring within 28 days after the first dose of mRNA vaccine) per 1 million individuals vaccinated in the study period was greater for CX-024414 compared to TOZINAMERAN. The SMR for myocarditis was >1 for both vaccines (showing excess risk), with the CX-024414 vaccine having a greater SMR than TOZINAMERAN. For pericarditis, the results showed an SMR > 1 when considering the lowest background incidence; however, when the highest background incidence was considered, the TOZINAMERAN vaccine showed an SMR < 1. In either case, the pericarditis SMRs for CX-024414 were greater than that for TOZINAMERAN. Similar results were observed for the disproportionality analysis.

In Germany, surveillance data from the Paul Ehrlich Institute reported a rate for myocarditis in men 18–29 years old of 11.71 cases per 100,000 COVID-19 Moderna vaccine recipients in German data and 7.79 in the Moderna Global Safety Database [29]. Furthermore, two other large European studies reported the occurrence of myocarditis and pericarditis after COVID-19 mRNA vaccination [29]. The first one, the Nordic cohort study [29] of 23 million residents of Denmark, Finland, Norway, and Sweden, observed that during the 28-day risk periods following vaccination, 1092 incident myocarditis cases and 1154 incident pericarditis cases occurred. Among them, 106 and 123 myocarditis cases occurred following first- and second-dose vaccinations with TOZINAMERAN, respectively, and 15 and 67 following CX-024414, respectively. The second one, the population-based case-control study in France [29], showed a large increase in the myocarditis odds within seven days after vaccination. In this study, the association with the risk of myocarditis appeared particularly pronounced in young men under 30 years of age, particularly after the second dose of the CX-024414 vaccine (odds ratio (OR) 79.8; 95% CI [29.8–213.4]). A Danish population-based cohort study observed that vaccination with the Moderna vaccine was significantly associated with an increased risk of myocarditis among 12- to 39-year-olds, while vaccination with the Pfizer-BioNTech vaccine was significantly associated with an increased risk among women [30].

Meanwhile, it is noteworthy to mention that the risk of myocarditis among adolescents was found in one study to be lower after a booster compared to the second dose of the TOZINAMERAN vaccine [31].

While an elevated risk of myocarditis following vaccination with mRNA COVID-19 vaccine was observed, the evidence regarding pericarditis remained equivocal. The population-based case-control study in France observed an increased risk of pericarditis in people under 30 years of age, in particular after the second dose in men (OR 15.0, 95% CI, 3.3–68.4) and after the first dose in women (OR 27.9, 95% CI, 2.4–328.0) [29], while the Danish population-based cohort study observed an increased risk of pericarditis among 12- to 39-years old [30]. On the other hand, Patone et al. [32] observed that there was no evidence of an increased risk of pericarditis following vaccination, except in 1 to 28 days after the second dose of the Moderna vaccine. Furthermore, Diaz et al. [16] reported that pericarditis affected older patients later, after either the first or second dose.

The elevated risk of myocarditis (and perhaps pericarditis) after receiving mRNA COVID-19 vaccine should be evaluated in light of the greater risk of myocarditis or pericarditis following SARS-CoV-2 infection [32]. Oberweis et al. [33] suggested that SARS-CoV-2 infection can cause cardiac injury directly (e.g., through angiotensin-converting enzyme binding in the heart) and indirectly through a toxic inflammatory reaction with cytokine storm, with the indirect mechanism being more frequent. Furthermore, the authors hypothesized that some children have died from COVID-19 because they may have been more susceptible to heart damage from the cytokine storm caused by COVID-19 than the standard respiratory distress syndrome seen in adults.

Even though the rare occurrence of myocarditis and pericarditis can be deadly, the majority of myocarditis cases linked to vaccines have been mild and self-limiting [25], whereas SARS-CoV-2 infection may carry a serious risk of morbidity and mortality over time, especially for the unvaccinated. Myocardial injury was highly common among hospitalized COVID-19 patients [34]. Patone et al. [32] emphasized that compared to the risk from COVID-19 vaccine, SARS-CoV-2 infection was associated with a significant increase in the risk of myocarditis, pericarditis, and cardiac arrhythmia-related hospitalization or death. Therefore, the benefits of SARS-CoV-2 mRNA vaccination that lowers both the risk of infection and the risk of hospitalization should be considered when interpreting the results of our study.

Furthermore, our results should be interpreted in light of the fact that myocarditis and pericarditis have also occurred following immunizations such as smallpox, influenza, hepatitis B, or other vaccines [1,2,3].

Our study is subject to certain limitations. First, in the EudraVigilance dataset, the information for the dose of the vaccine is missing for the majority of data entries. Therefore, we used all the count of the myocarditis or pericarditis ADRs reported in the study period in the respective equation for the calculation of myocarditis or pericarditis reporting rates. Secondly, we cannot directly compare the reporting rate of myocarditis or pericarditis to the incidence rate in the general EU/EEA population because they represent different measures that use different definitions of the time at risk. Consequently, whether the incidence of myocarditis or pericarditis is higher in vaccinated people than in the general population is beyond the scope of this study. Since we do not know the exact background incidence rate, which may vary substantially among different vaccine group populations, as well as knowing that the data were based on different assumptions [35], the findings should be interpreted with caution. Thirdly, we cannot exclude the possibility of ADRs’ registration underestimation. There is a possibility of underreporting myocarditis and pericarditis, which can impose non-differential misclassification. Fourthly, we were unable to calculate myocarditis and pericarditis incidence for each vaccine that is adjusted for demographics and other factors because the information on confounding is absent; except for sex and age group, information on other variables was missing. Fifthly, data on the vaccination rate stratified by age and sex are absent and the reporting rates were not standardized for age due to the unavailability of the data; therefore, we were unable to perform a stratified OE analysis. A vaccine’s safety profile may vary depending on the target population (e.g., higher risks in the youngest age groups); therefore, comparing reporting rates for age groups or countries should be avoided because it would introduce biases and inaccuracies.

## 5. Conclusions

To conclude, our study analysis supports an excess risk of myocarditis following the first dose of the mRNA COVID-19 vaccine, but the direction of the relationship between pericarditis and mRNA COVID-19 vaccine remains unclear. Future research is needed to calculate age and sex standardized reporting rates, stratified OE analyses, as well as analyses following the second or more doses of mRNA COVID-19 vaccine. The benefits of COVID-19 vaccination exceed the risks of adverse events; however, pharmacovigilance methods continue to play a pivotal role in monitoring, identifying, and reducing ADR risk.

## Figures and Tables

**Table 1:** All myocarditis ADRs reported from 1 January 2021 to 11 February 2022 stratified by age group and sex.

Vaccine Name, *n* of Total Cardiac ADRs (1 January 2021–11 February 2022)	Patient Age Group	Patient Sex			Myocarditis ADRs
		Female	Male	NS	*n* (%)
Moderna (CX-024414) N = 9078	12–17 Years	5	67	0	72 (5.67)
	18–64 Years	231	883	6	1120 (88.26)
	65–85 Years	29	27	0	56 (4.41)
	More than 85	2	1	0	3 (0.24)
	Not Specified	6	10	2	18 (1.42)
	Total (%)	273 (21.51)	988 (77.86)	8 (0.63)	1269 (100)
Pfizer-BioNTech (TOZINAMERAN) N = 38,297	5–11 Years	3	4	0	7 (0.17)
	12–17 Years	65	492	5	562 (13.75)
	18–64 Years	897	2187	22	3106 (76.00)
	65–85 Years	122	122	2	246 (6.02)
	More than 85	11	12	0	23 (0.56)
	Not Specified	40	85	18	143 (3.50)
	Total (%)	1138 (27.84)	2902 (71.01)	47 (1.15)	4087 (100)

Abbreviations: ADRs, adverse drug reactions; *n*, number; NS, not specified.

**Table 2:** All pericarditis ADRs reported from 1 January 2021 to 11 February 2022 stratified by age group and sex.

Vaccine Name, *n* of Total Cardiac ADRs (1 January 2021–11 February 2022)	Patient Age Group	Patient Sex			Pericarditis ADRs
		Female	Male	NS	*n* (%)
Moderna (CX-024414) N = 9078	12–17 Years	5	7	0	12 (1.67)
	18–64 Years	266	336	4	606 (84.52)
	65–85 Years	46	43	0	89 (12.41)
	More than 85	2	0	0	2 (0.28)
	Not Specified	4	4	0	8 (1.12)
	Total	323 (45.05)	390 (54.39)	4 (0.56)	717 (100)
Pfizer-BioNTech (TOZINAMERAN) N = 38,297	12–17 Years	49	89	0	138 (4.92)
	18–64 Years	1157	1068	10	2235 (79.74)
	65–85 Years	160	184	0	344 (12.27)
	More than 85	15	25	0	40 (1.43)
	Not Specified	24	20	2	46 (1.64)
	Total	1405 (50.12)	1386 (49.45)	12 (0.43)	2803 (100)

Abbreviations: ADRs, adverse drug reactions; *n*, number; NS, not specified.

**Table 3:** The severity of the ADRs reported from 1 January 2021 to 11 February 2022 stratified by the mRNA COVID-19 vaccines. The ADR categories are not mutually exclusive. Some cases might belong to 2 or more categories. Thus, the row sum is greater than the total number of observations.

ADR Reported by the Type of mRNA COVID-19 Vaccines		Requires Hospitalizations or Prolongation of Hospitalization *n* (%)	Other Medically Important Information *n* (%)	Life Threatening *n* (%)	Non-Serious *n* (%)	Results in Disability or Incapacity *n* (%)	Results in Death *n* (%)
Myocarditis	Moderna (CX-024414) N = 1269	977 (76.99)	277 (21.83)	138 (10.88)	40 (3.15)	34 (2.68)	7 (0.55)
	Pfizer-BioNTech (TOZINAMERAN) N = 4087	2573 (62.96)	1224 (29.95)	454 (11.11)	111 (2.72)	114 (2.79)	37 (0.91)
Pericarditis	Moderna (CX-024414) N = 717	326 (45.47)	325 (45.33)	35 (4.88)	75 (10.46)	11 (1.53)	4 (0.56)
	Pfizer-BioNTech (TOZINAMERAN) N = 2803	1190 (42.46)	1344 (47.95)	165 (5.89)	296 (10.56)	85 (3.03)	9 (0.32)

**Table 4:** Observed-to-expected analysis and Information Component for myocarditis.

Vaccine Name	Person-Time at Risk (100,000 Person-Years)	Background Incidence (Lowest, Highest)	Expected Myocarditis	Observed Myocarditis	SMR (95% CI)	IC (95% CI)
Moderna (CX-024414)	56.32	1	56.32	1269	22.53 (21.31–23.81)	4.48 (4.39–4.55)
		10	563.2		2.253 (2.13–2.38)	1.17 (1.08–1.24)
Pfizer-BioNTech (TOZINAME RAN)	371.35	1	371.35	4087	11.01 (10.67–11.35)	3.46 (3.41–3.50)
		10	3713.5		1.10 (1.07–1.14)	0.14 (0.09–0.18)

Abbreviations: SMR, standardized morbidity ratio; IC, information component; CI, confidence interval.

**Table 5:** Observed-to-expected analysis and Information Component for pericarditis.

Vaccine Name	Person-Time at Risk (100,000 Person-Years)	Background Incidence (Lowest, Highest)	Expected Pericarditis	Observed Pericarditis	SMR (95% CI)	IC (95% CI)
Moderna (CX-024414)	56.32	1	56.32	717	12.73 (11.82–13.70)	3.66 (3.54–3.75)
		10	563.2		1.27 (1.18–1.37)	0.35 (0.23–0.44)
Pfizer-BioNTech (TOZINAME RAN)	371.35	1	371.35	2803	7.55 (7.27–7.83)	2.91 (2.85–2.96)
		10	3713.5		0.75 (0.73–0.78)	−0.41 (–0.47––0.36)

Abbreviations: SMR, standardized morbidity ratio; IC, information component; CI, confidence interval.

## Data Availability

Publicly available datasets were analyzed in this study. These data can be found here: https://www.adrreports.eu/ (accessed on 16 February 2021) and https://www.ecdc.europa.eu/en/publications-data/data-covid-19-vaccination-eu-eea (accessed on 16 February 2021).
